# Healthy physical education curriculum model and students’ extracurricular sports participation ——test based on the trans-contextual model of motivation

**DOI:** 10.1186/s12889-022-14483-0

**Published:** 2022-11-15

**Authors:** Xiaoliang Hao, Yunyun Yang

**Affiliations:** 1 College of Physical Education, Taiyuan University of Technology, 79 Yingze West Street, Taiyuan City, 030024 Shanxi Province China; 2 College of Electrical and Power Engineering, Taiyuan University of Technology, 79 Yingze West Street, Taiyuan City, 030024 Shanxi Province China

**Keywords:** Physical activity, Health, Transcontextual model, Motivation

## Abstract

**Background:**

In recent years, the mental health level and physical activity level of Chinese teenagers are not ideal, and all sectors of society are actively reversing this bad situation. The purpose of this study is to test the influence of healthy physical education curriculum model on middle school students’ extracurricular sports participation based on the trans-contextual model of motivation (TCM).

**Methods:**

The trial adopts quasi-experimental design comparing equivalent groups. The experimental group adopted the healthy physical education curriculum model in physical education (PE), and the control group adopted the technical-traditional teaching. During the 12 weeks intervention, 327 junior school freshmen completed the test of TCM variables four times as the pre-test, test 2, test 3 and post-test of this experimental study.

**Results:**

After the intervention, students’ perceived need support in PE, autonomous motivation in PE, autonomous motivation in leisure time (LT)and the amount of extracurricular sports activities in the experimental group have increased significantly. The perceived need support of experimental group students can predict autonomous motivation in PE positively (*β* = 0.385, *P*<.001); Autonomous motivation in PE can predict autonomous motivation in LT positively (*β* = 0.462, *P*<.001); Autonomous motivation in LT can predict the intention of extracurricular sports participation positively, and the direct effect was significant (*β* = 0.172, *P*<.01), the total indirect effect was significant (*β* = 0.382, *P*<.001), the indirect effect of subjective norms was not significant (*P*>.05); Extracurricular sports participation intention can predict the amount of extracurricular sports activities positively (*β* = 0.327, *P*<.001).

**Conclusions:**

The structural characteristics of healthy physical education curriculum model provide need support for students’ learning, improve students’ autonomous motivation in and out of PE, and finally promote students’ participation in extracurricular sports.

## Introduction

In recent years, the level of health of Chinese students has been criticized by all sectors of society. Although the level of health of teenagers is affected by many factors, most of their time is spent in school, so the physical education curriculum should bear some responsibility. For a long time, the lack of guiding ideology and the perplexity practical factors have led to the lack of guarantee of the quantity and quality of physical education. The graduation grades of most schools and schools with insufficient conditions even have the dilemma of “absence” of physical education, even if physical education can be carried out normally, “students don’t sweat” is also the teaching normality [1]. Under the far-reaching influence of the teaching thought of the former Soviet Union, China’s PE teachers uphold the “sports technology center theory” in the class, the teaching closely focuses on teachers and single technical action, students only need to constantly imitate the single technical action taught by teachers until dynamic shaping. However, such teaching results are: Students have received physical education for 12 years and can hardly master a sport completely; Students like sports but don’t like physical education class.

On the basis of long-term theoretical research and practical exploration, professor Liu Ji formally put forward the healthy physical education curriculum model in 2015 [2]. Healthy physical education curriculum model clearly puts forward the implementation standards of the key points of teaching: In terms of sports load, the average heart rate of students in class should reach 140–160 beats/min, and students’ continuous exercise time should account for about 75% of the total class time. It should be noted that the practice time of each student is not required to reach 75% of the total class time, but the teacher arranges the time of the whole class in the static state to not exceed 25% of the total class time as far as possible due to the reasons of team formation, centralized explanation and demonstration. In terms of sports skills, no matter the new teaching or review class, it emphasizes focusing on activities and competitions, abandoning teaching only around a single skill whole class, and the time is maintained at about 20 min. In terms of physical exercise, each class is equipped with special physical exercise links, and pay attention to the creation of exercise situations to comprehensively develop students’ physical fitness, with a time of about 10 minutes. The structural characteristics of healthy physical education curriculum model not only help students master a sport, but also meet students’ learning needs.

Physical education and extracurricular sports constitute all the sources of students’ physical exercise. Extracurricular sports is not only the extension of physical education, but also the destination of PE. The ideas and methods of healthy physical education curriculum model are also applicable to students’ extracurricular physical exercise, that is, as long as students can grasp the three key points of healthy physical education curriculum model in the process of extracurricular sports participation, they can improve their health level. As we all know, the goal of Chinese current physical education curriculum is to cultivate students’ core literacy. In fact, it is not enough to achieve the goal only by limited amount of PE teaching, which requires the active combination of family, school and society to ensure students’ extracurricular sports participation [3]. Only by screwing PE and extracurricular sports into a rope, can we work together to achieve the goal of physical education curriculum.

**Fig. 1:**
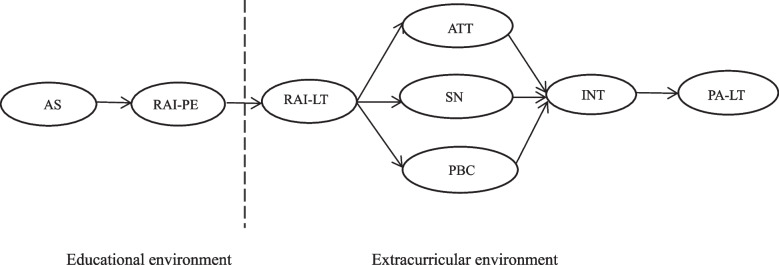
Sketch map of trans-contextual model of motivation. Note: AS, perceived autonomous support in PE; RAI-PE, autonomous motivation index of PE；RAI-LT, autonomous motivation index of leisure time; ATT, attitude; SN, subjective norm; PBC, perceived behavioral control; INT, extracurricular sports participation intention; PA-LT, the amount of extracurricular sports activities

Nowadays, the lack of physical activity has become a global problem of students. Therefore, experts and scholars try to use various psychological models to explore the key factors affecting teenagers’ participation in sports activities, and try to intervene through practice. In recent years, foreign countries have widely used the theoretical framework of multiple integration to verify the transfer process of motivation and behavior in similar educational environment, this theoretical framework of multiple integration is the trans-contextual model of motivation [4]. TCM was put forward by professor Hagger of health psychology and behavioral medicine at Curtin university, Australia. TCM points out the whole process of motivation transfer from one environment to another similar environment, it believes that students’ perceived autonomous support and autonomous motivation in the class are related to the autonomous motivation of extracurricular related activities, and describes the relationship between autonomous motivation of extracurricular activities and belief based structure, intention to participate in extracurricular activities and actual participation behavior. It is worth mentioning that the model has been widely used in physical education and extracurricular sports environment since it was proposed. The whole process is shown in Fig. 1.

### Study design

Before the experimental research, the teacher of the experimental group was trained in the theory and practice of the healthy physical education curriculum model, and achieved satisfactory training results. According to the school facilities, teachers’ characteristics and students’ learning experience, the teaching experiment themes are: long jump and rope skipping. Among them, long jump teaching lasts for 6 weeks and rope skipping teaching lasts for 6 weeks. Three classes a week, each class lasting 40 minutes.

The experimental group firmly grasped the three key points of the healthy physical education curriculum model, and the teaching plan was jointly written between researcher and teacher after discussion. The control group implements routine teaching, that is, technical-traditional teaching, has the following remarkable characteristics: The average heart rate of students in the whole class is less than 140 beats/min; Mainly adopts single movement skill teaching; There is no special physical exercise in the class. During the 12 weeks intervention period, all students completed the test of TCM variables four times as the pre-test, 2-test, 3-test and post-test of the experimental research.

During the experiment, try to avoid the influence of irrelevant factors on the results, the control process is mainly reflected in the following aspects: The amount of teaching time of two groups must be consistent; If the teaching experiment cannot be carried out normally due to weather and various school activities, the amount of teaching are required to make up in combination with the situation of school and students；Set the same teaching theme to avoid the interference of different themes on the experimental results；The gender of the teachers in the experimental group and the control group are the same, close in age；No matter in the experimental group or the control group, students who have been absent from class for 3 times or more will not participate in various tests of the experimental research.

### Participants

As a native of Shanxi province, the author is familiar with schools in the region, so this teaching experiment is conducted in schools in Shanxi province. Junior school freshmen have no enrollment pressure, and basically have the ability to fill in the questionnaire, therefore, junior school freshmen are selected as the experimental samples. In view of this, cluster sampling was adopted, and three classes of grade one of junior high school were selected in the experimental school and the control school respectively. Since the subsequent structural equation model requires that the number of samples should not be less than 150, this is the reason why the two groups of students choose three classes, the number of samples is as follows: 165 students in the experimental group (83 boys and 82 girls) and 162 students in the control group (83 boys and 79 girls). Students with past medical history, cardiovascular diseases and family genetic diseases will not participate in this teaching experiment. In the process of recruiting subjects, there were no cases of refusing to participate, and all subjects completely participated in the teaching experiment.

### Test variables

#### Perceived need support in PE

To meet the basic psychological needs of students in the class, teachers need to provide supportive teaching, including autonomous support, structural support and interpersonal involvement, only when the above three supporting conditions are met at the same time can the teaching effect be optimized to the greatest extent [5, 6]. However, most of the existing studies only start from the dimension of autonomous support, in view of this, this study follows the academic view of scholar Long Yin and modifies the independent variable “perceived autonomous support” of TCM to “perceived need support” covering the three dimensions of supportive teaching. The scale of perceived need support in PE was compiled by Chinese scholar Long Yin [7]. The scale covers three dimensions: autonomous support, capability support and relationship support. Autonomous supported testing, Long Yin combined with health care climate questionnaire, which is modified into the autonomous support questionnaire for physical education, including 6 items. The questionnaire prepared by Standage is used to evaluate students’ capability support and relationship support. Capability support covers 4 items and relationship support covers 5 items. Through the strict scale preparation procedure, Long Yin integrated the three dimensions into the scale of perceived need support in PE. The new scale adopts likert level 7 scoring, and 1–7 scores are calculated from low to high according to the recognition degree of the topic, such as “I think the physical education teacher provides me with a lot of choices in the class”.

### Autonomous motivation in PE

The cause perception scale translated and revised by Boguang Zhong of Hong Kong Baptist University is used to measure students’ autonomous motivation in PE [8]. The scale takes middle school students as the research subject and covers five dimensions, namely, no motivation, external regulation, introjected regulation, identity regulation and internal regulation, each dimension includes three items. Likert level 7 scoring is adopted for the scale, and 1–7 scores are given according to the degree of agreement with the topic from low to high, such as “because I like learning new motor skills”. Based on the practice of Vallerand and Standage [9], the level of personal autonomous motivation is calculated by using the following formula: 2 × internal regulation + identity regulation - introjected regulation - 2 × external regulation, the higher the score, the more internal the motivation.

### Autonomous motivation in leisure time

Behavioral regulation in exercise questionnaire (breq-2), compiled by Markland, is the most widely used tool to measure exercise behavior regulation. Hong Kong scholar Jingdong Liu, revised the scale and finally formed the Chinese version of exercise behavior regulation scale [10]. The revised scale adopts likert level 5 scoring, and 0–4 points are calculated from low to high according to the recognition of the topic, such as “I exercise because others say I should exercise”. As mentioned earlier, the personal motivation level is calculated by using the formula based on the practice of Vallerand and Standage [9]: 2 × internal regulation + identity regulation - introjected regulation - 2 × external regulation, the higher the score, the more internal the motivation.

### Theory of planned behavior

This study adopts the Chinese version of the planned behavior theory scale of professor Lijuan Wang of Shanghai Institute of physical education [11]. The scale takes middle school students as the research subject and covers four dimensions: behavior intention, exercise attitude, subjective norms and perceived behavior control. The scale adopts likert level 7 scoring, such as “I plan to exercise at least three times in my spare time next week”. According to the research needs, the exercise behavior predicted in the original scale in the next 7 days is modified to predict the exercise behavior in the next month, such as “I plan next week...” is changed to “Next month, I plan every week...”. In addition, considering that a single measurement model has only one observation variable, that is, only one item in a single dimension will make the model unrecognizable [12], the single item of subjective norms dimension “Important people around me (parents, friends and teachers) think I should exercise at least three times in my spare time next week” is divided into three items, such as “My parents think I should exercise at least three times a week in my spare time next month”.

### Amount of extracurricular sports activities

Deqing Liang ‘s physical activity rating scale was used to measure the amount of students’ extracurricular sports activities [13]. The scale examines the amount of exercise from three aspects: intensity, time and frequency of physical exercise. The calculation formula is: Amount of exercise = intensity × time × frequency. The intensity and frequency are recorded as 1–5 points from 1 to 5 levels, and the time is recorded as 0–4 points from 1 to 5 levels. The highest score is 100 points and the lowest score is 0 points.

### Statistical analysis

Using independent sample t test in SPSS software (IBM Corp, Armonk, NY, USA) to examine the homogeneity of pre-test values of TCM variables of students in the experimental group and control group. Using repeated measurement ANOVA to compare the differences of TCM variables between two groups of students at different times; In order to explore the influence process of healthy physical education curriculum model on students’ extracurricular sports participation results, the relationship between latent variables of TCM needs to be established. First, using the syntax programming of Mplus7.4 software (Linda Muthén & Bengt Muthén, USA) to estimate the measurement model of each latent variable of TCM, so as to obtain the item reliability of the observed variables in each latent variable. Then, based on the item reliability, the combined reliability and aggregate validity of each latent variable are derived by using the formula. The correlation between latent variables is also obtained through Mplus software (adding an instruction “TECH4” to the “OUTPUT” syntax). Only when the combined reliability, aggregate validity and differential validity of each latent variable are within an acceptable range can we explore the relationship between latent variables. Last, the direct and indirect relationship between TCM latent variables are clarified by path analysis of structural equation model of Mplus7.4 software.

## Results

The subjects participating in this experimental study were junior school freshmen, 165 students in the experimental group (83 boys and 82 girls), and 162 students in the control group (83 boys and 79 girls). The students of each class are shown in Table 1.

**Table 1:** Descriptive statistics of the students of each class

Class	Male	Female	Age
Experimental class 1	28	28	13.44 ± 0.53
Experimental class 2	27	26	13.39 ± 0.69
Experimental class 3	28	28	13.62 ± 0.51
Control class 1	30	25	13.21 ± 0.65
Control class 2	26	27	13.74 ± 0.42
Control class 3	27	27	13.09 ± 0.47

The two groups of boys’ pretest of perceived need support were tested by independent sample t-test, and the results showed that there was no significant difference(*t* = − 0.996, *P*>.05). The two groups of girls’ pretest scores were tested by independent sample t-test, and the results showed that there was no significant difference(*t* = 0.308, *P*>.05). Then, repeated measurement analysis of variance was carried out for students’ perceived need support. The inter group factor was group, the intra group factor was measurement time, and the interaction was group*time. The spherical test showed *P*<.05, and the analysis results were subject to multivariate test. The main effect of time was significant (*F* = 451.824, *P*<.001), and the partial ETA square was 0.809, that is, with the passage of time, no matter the experimental group or the control group, there are significant differences in their perceived need support. The interaction of time*group was significant (*F* = 243.875, *P*<.001), and the partial ETA square was 0.694, so a simple effect analysis was needed. The syntax is: /EMMEANS = TABLES(time*group)COMPARE(time)ADJ(SIDAK),/EMMEANS=.

**Fig. 2:**
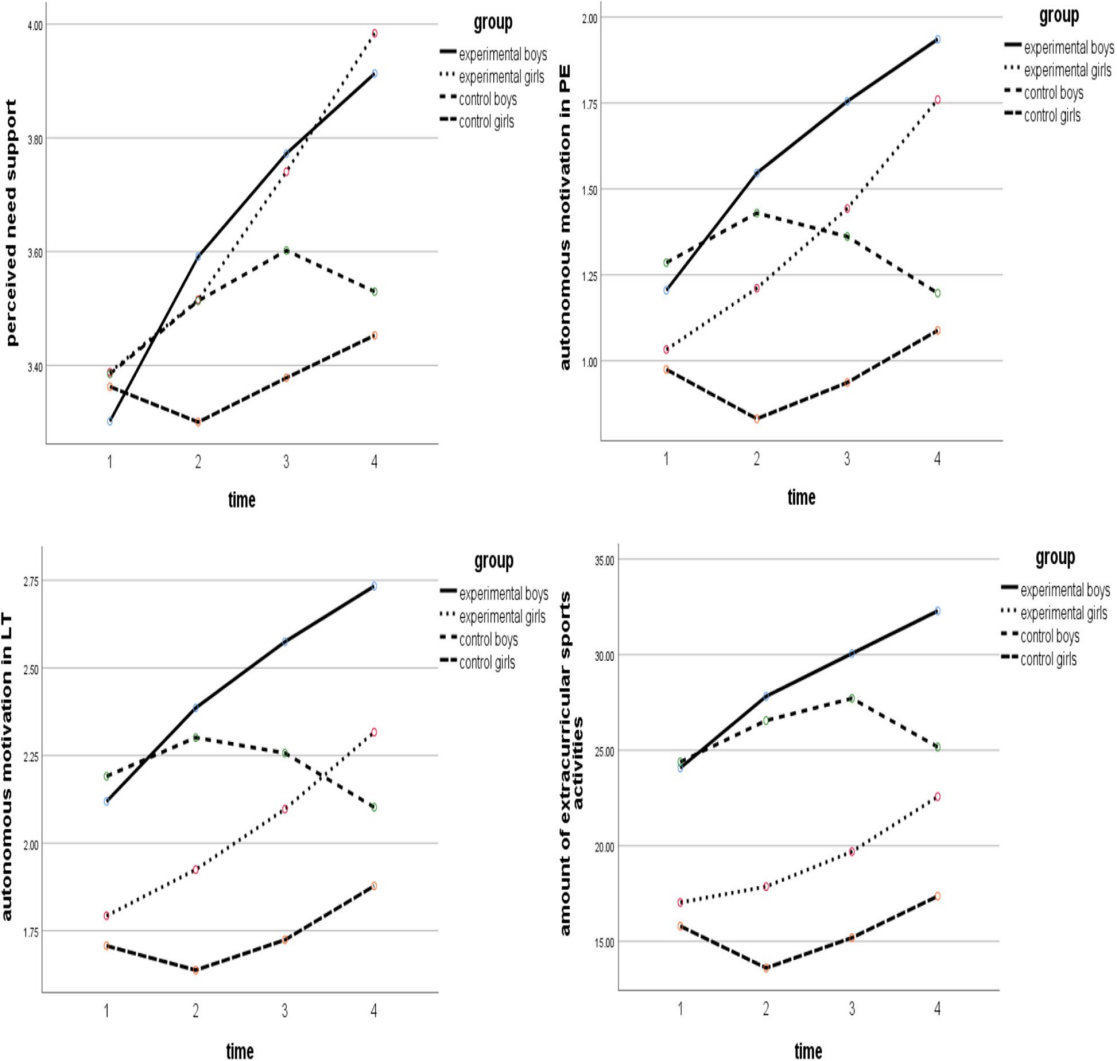
During the intervention, change diagram of students at different time of perceived need support, autonomous motivation in PE, autonomous motivation in LT and the amount of extracurricular sports activities

TABLES(time*group)COMPARE(group)ADJ(SIDAK). The simple effect results showed that there was significant difference in the perceived need support between the two groups of boys’ in the test 4 only, however, the boys in the experimental group made significant progress in test 1 to 2, test 2 to 3, test 3 to 4. In contrast, the change of test 3 to 4 of the boys in the control group showed a downward trend; The scores of girls in the experimental group are significantly higher than those in the control group, and the mean difference gradually expands with the passage of time, followed by test2 (Δ = 0.215), test3 (Δ = 0.362), test4 (Δ = 0.531). In addition, girls in the experimental group made significant progress in test 1 to 2, test 2 to 3, test 3 to 4, while girls in the control group showed a downward trend in the change of test 1 to 2. Overall, during the intervention period, the progress of boys in the experimental group was Δ = 0.611, the progress of girls in the experimental group was Δ = 0.596. The following is the change chart of perceived need support of junior school freshmen. In the same way, before and after the intervention, the changes of autonomous motivation in PE, autonomous motivation in LT and the amount of extracurricular sports activities of junior school freshmen are shown in Fig. 2.

From the above, it can be seen that the teaching of 12 weeks healthy physical education curriculum model has effectively improved students’ learning results in and out of PE. In order to explore the causal relationship between TCM variables of students in the experimental group, we next obtained it through structural equation model path analysis.

There are many latent variables involved in structural equation modeling, and some latent variables may contain several latent variables, the increase of indicators will complicate the model and is not conducive to building the relationship between key latent variables. Therefore, researchers often use the item packing method to convert some latent variables into explicit variables, that is, calculate the total score of its observed variables, and then take the mean score, so as to purify the measurement error, simplify the model, and finally improve the parameter estimation and model fitting [14]. The structural model of this study is relatively complex, therefore, the three latent variables contained in the perceived need support, autonomous support, capability support and relationship support are transformed into explicit variables through item packing method. Similar practices include the five latent variables contained in autonomous motivation in PE and the five latent variables contained in autonomous motivation in LT, etc. We know that to establish the relationship between structural models, the primary task is to do a good job in each measurement model [15, 16], however, considering that the purpose of this study is to obtain the direct and indirect relationship between TCM latent variables, the author does not depict the result diagram of each measurement model, but lists the construction validity of the measurement models as shown in Table 2.

**Table 2:** Reliability, aggregate validity and discriminant validity of TCM variables

Dimension	Item	IR	CR	AVE	Discriminant Validity							
					NS	RAI-PE	RAI-LT	INT	ATT	SN	PBC	PA-LT
NS	3	0.713–0.824	0.793	0.632	**0.795**							
RAI-PE	5	0.627–0.786	0.705	0.588	0.346	**0.767**						
RAI-LT	5	0.633–0.776	0.716	0.539	0.253	0.409	**0.734**					
INT	3	0.766–0.835	0.783	0.615	0.217	0.208	0.212	**0.784**				
ATT	3	0.685–0.769	0.789	0.603	0.198	0.188	0.345	0.510	**0.777**			
SN	3	0.574–0.718	0.634	0.472	0.121	0.087	0.127	0.137	0.219	**0.687**		
PBC	3	0.671–0.739	0.675	0.501	0.169	0.197	0.357	0.485	0.446	0.266	**0.709**	
PA-LT	3	0.675–0.820	0.692	0.577	0.152	0.211	0.367	0.382	0.385	0.114	0.347	**0.760**

*IR* item reliability, *CR* composite reliability, *NS* perceived need support in PE; According to Fornell and larcker’s recommendations [17], the bold numbers in the table are the square root of AVE (mean variance extraction), and the lower triangle are the pearson correlation between dimensions

It can be seen from the above that the item reliability of the eight latent variables basically meet the recommended value, the composite reliability also meet the standard, and the aggregate validity also within the acceptable range, in addition, there are discriminant validity between dimensions, which are suitable for further analysis of the structural model.

**Fig. 3:**
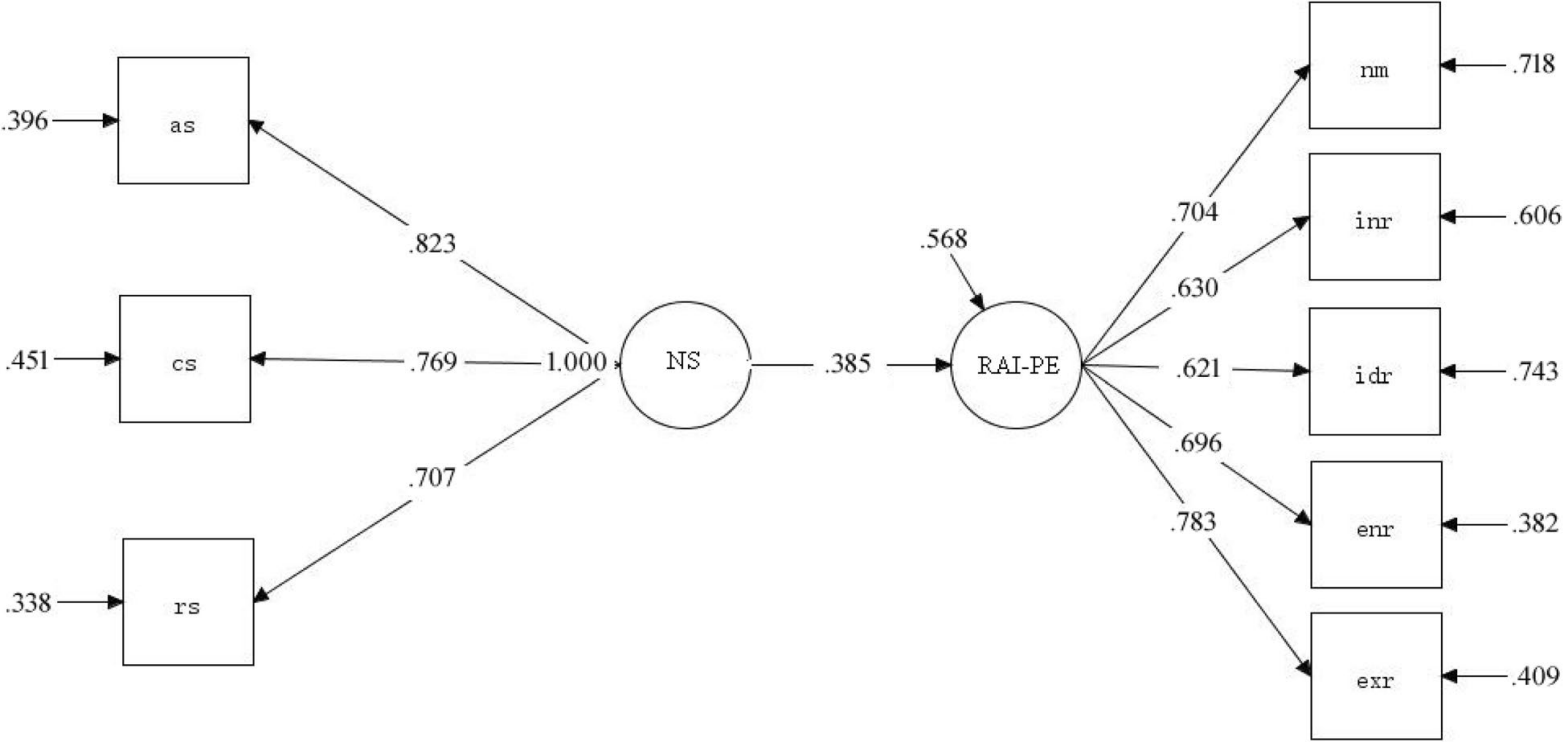
Structural model of perceived need support in PE and autonomous motivation in PE of experimental group students

Among the many latent variables of TCM, the first thing to be analyzed is the relationship between the perceived need support and autonomous motivation in PE. Edit the measurement models of NS and RAI-PE under the “MODEL” command in the Mplus home panel, that is, “NS BY as cs rs” (three observed variables of NS); “RAI-PE BY nm inr idr enr exr” (five observed variables of RAI-PE). Then edit the structural model, “RAI-PE ON NS”. Finally, the program is calculated. The path coefficient of the simple structure model takes the standardized value. The results show that students’ perceived need support can positively predict autonomous motivation in PE (*β* = 0.385, *P*<.001), and the *R*^*2*^ value of autonomous motivation in PE is 0.41, indicating that the model has reached a medium level of explanatory effect, that is, students’ perceived need support explains 41% of the variation of autonomous motivation in PE. The impact process is shown in Fig. 3.

**Fig. 4:**
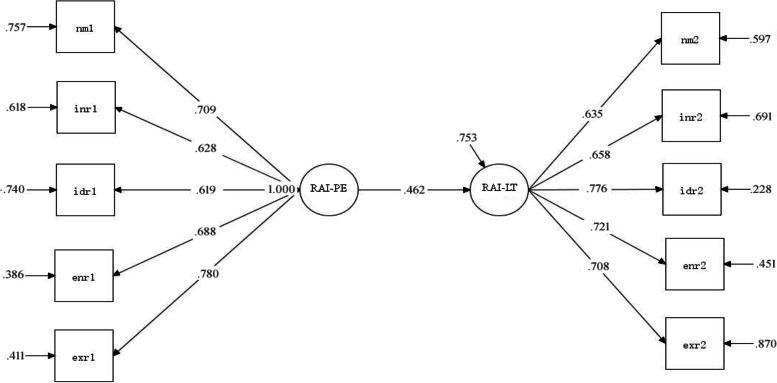
Structural model of autonomous motivation in PE and autonomous motivation in LT of experimental group students

The results of the standardized model show that autonomous motivation in PE can positively predict autonomous motivation in LT (*β* = 0.462, *P*<.001), *R*^*2*^ value is 0.40, that is, autonomous motivation in PE explains 40% of the variation of autonomous motivation in LT. The impact process is shown in Fig. 4.

**Fig. 5:**
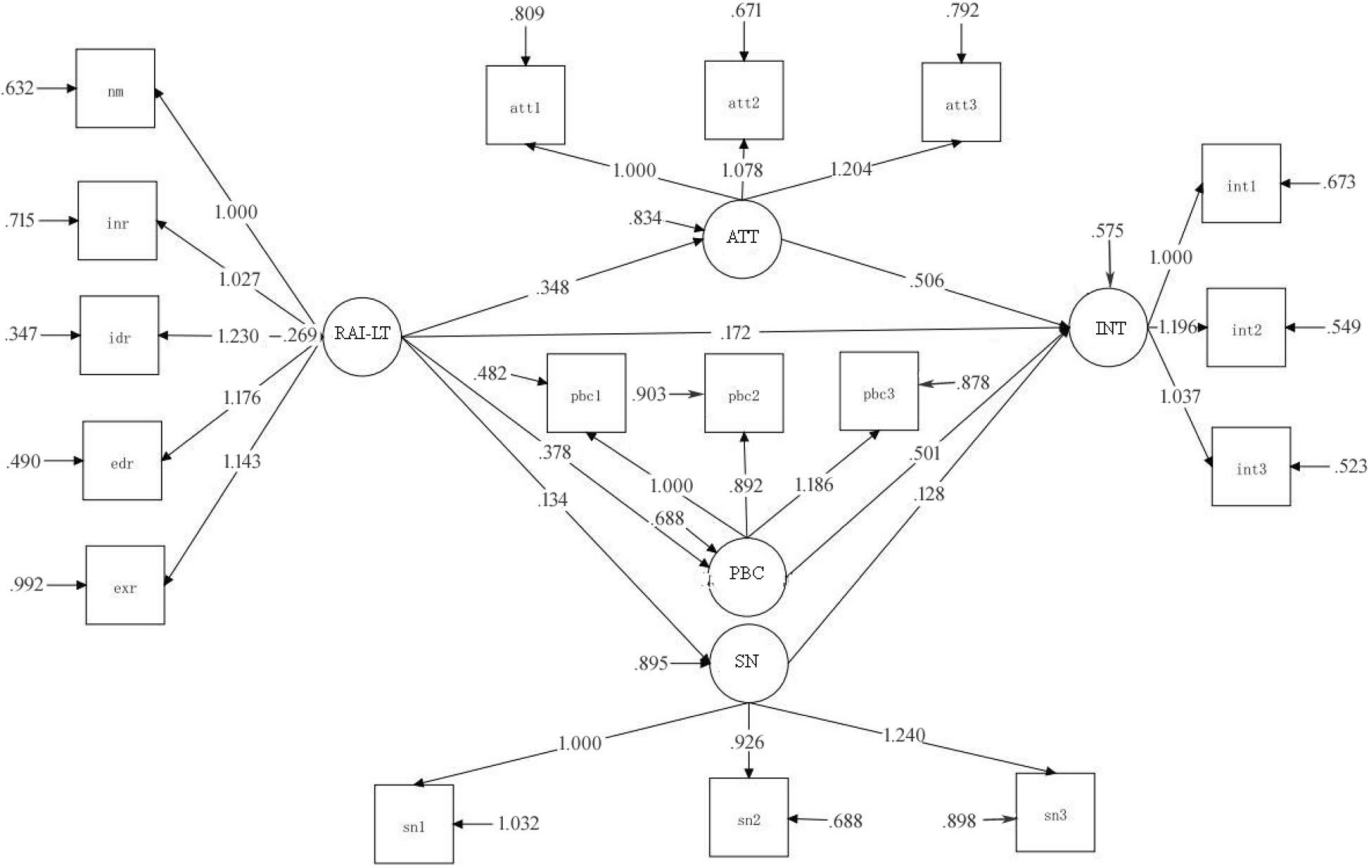
Mediation model between autonomous motivation in LT and extracurricular sports participation intention of experimental group students

In order to clarify the direct and indirect relationship between RAI-LT and INT, it is necessary to conduct path analysis on this intermediary model. Different from the simple path analysis, the path coefficient of the latent variables in the multiple mediation model take non-standard value. Add an instruction for analyzing indirect effects in the home panel: “MODEL INDIRECT”, then input syntax “INT IND RAI-LT” and perform operation. The results show that the indirect effect of path RAI-LT → ATT → INT was significant (*β* = 0.176, *P*<.01), the indirect effect of path RAI-LT → SN → INT was not significant (*β* = 0.017, *P*>.05), and the indirect effect of path RAI-LT → PBC → INT was significant (*β* = 0.189, *P*<.01). In addition, the total indirect effect of the model was significant (*β* = 0.382, *P*<.001), and the direct effect was also significant (*β* = 0.172, *P*<.01). In order to further test the difference of the three indirect paths, “DIFF12”, “DIFF13”, “DIFF23” are edited under the sub instruction of “MODEL CONSTRAINT”. The results show that the effect difference between path 1 and 2, path 2 and 3 (i.e. F1-F2, F2-F3) were significant (*P*<.05), and the effect difference between path 1 and 3 (i.e. F1-F3) was not significant (*P*>.05). The confidence intervals of deviation corrected bootstrap and percentile bootstrap are consistent, which shows the above research results. The results of the mediation model are shown in Fig. 5 and Table 3.

**Table 3:** Effects of autonomous motivation in LT on extracurricular sports participation intention and comparison of mediating effects of experimental group students

Path	Point estimate	Product of coefficients			BOOTSTRAP		95%CI	
					Bias-corrected confidence interval		Percentile confidence interval	
		Standard error	*Z* value	*P* value	lower	upper	lower	upper
Model effect								
RAI-LT → ATT → INT	0.176	0.070	2.514	**	0.058	0.540	0.069	0.552
RAI-LT → SN → INT	0.017	0.016	1.077	0.167	−0.044	0.349	−0.056	0.332
RAI-LT → PBC → INT	0.189	0.069	2.739	**	0.077	0.418	0.092	0.436
TOTAL INDIRECT	0.382	0.121	3.156	***	0.411	1.749	0.430	1.765
DIRECT	0.172	0.059	2.915	**	0.049	0.588	0.055	0.594
Comparison of multiple mediating effects								
F1-F2	0.159	0.062	2.570	0.022	0.011	0.235	0.026	0.253
F1-F3	−0.013	0.034	−0.377	0.480	−0.088	0.417	− 0.065	0.432
F2-F3	−0.172	0.065	−2.630	0.019	−0.589	−0.044	− 0.602	−0.060

*** *P*<.001, ** *P*<.01

**Fig. 6:**
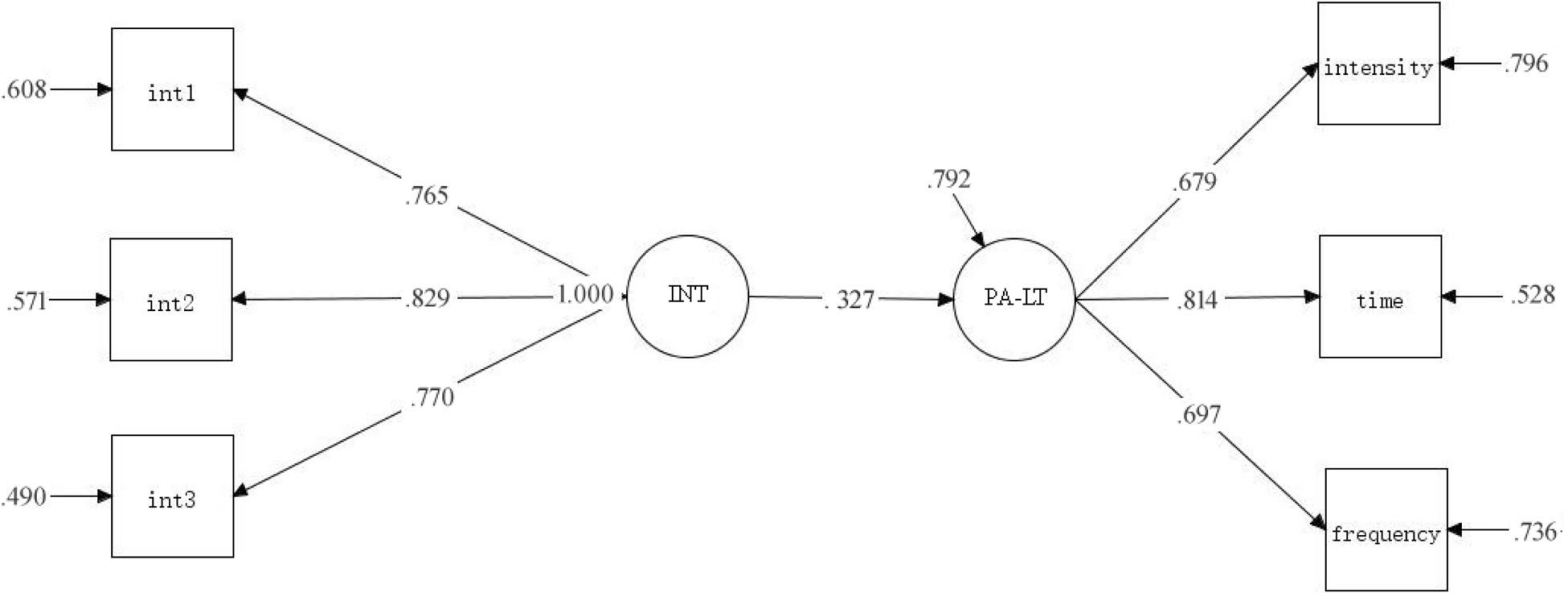
Structural model of extracurricular sports participation intention and the amount of extracurricular sports activities of experimental group students

The results of the standardized model show that the intention of extracurricular sports participation can positively predict the amount of extracurricular sports activities (*β* = 0.327, *P*<.001), *R*^*2*^ value was 0.34, which was intention explain 34% of the variation of behavior. The impact process is shown in Fig. 6.

## Discussion

### Differences in TCM variables between the two groups of students

#### Perceived need support

Boys in the experimental group made significant progress in all stages, and boys in the control group showed a downward trend in the change of test 3 to 4. The possible reason is that the teaching theme in the later stage of the experiment is rope skipping, and the teacher in the control group only teach single and double rope skipping around a short rope, obviously, such teaching did not take into account the lively characteristics of boys, the teacher of the experimental group better met students’ learning needs by creating various forms of rope skipping activities and competitions, as well as interesting and rich physical exercises. The girls in the experimental group have made significant progress in all stages, in contrast, the changes of girls from test 1 to 2 in the control group show a downward trend. The possible reason is that the teaching theme in the early stage of the experiment is long jump, and girls are naturally lack of interest in the long jump, in addition, the teacher in the control group carry out a large number of decomposition technical action teaching around the theme, while the teacher in the experimental group pay more attention to let the students consolidate the technical details through the complete long jump.

#### Autonomous motivation in PE

The boys in the experimental group made progress at all stages. In contrast, the change of boys in the control group from test 2 to 3, test 3 to 4 showed a downward trend. For boys in grade one of junior high school, they may prefer long jump to rope skipping, in addition, the teacher of control group lack organizational design in rope skipping teaching, which must be difficult to stimulate boys’ learning motivation. The girls in the experimental group made progress at all stages, but the changes from test 1 to 2 of girls’ in the control group showed a downward trend. The possible reason is that girls originally lack interest in the long jump, while the control group teacher failed to create learning situations for girls, resulting this decline.

#### Autonomous motivation in LT

The students in the experimental group made remarkable progress at all stages. In contrast, boys in the control group showed a downward trend in test 2 to 3, test 3 to 4,while girls in the control group made no significant progress in test 2 to 3 and made a step backward in test 1 to 2. Study found that the score change of boys and girls in control group show almost opposite trend, combined with teaching theme, it is speculated that students’ preference for teaching theme may lead to the difference of extracurricular sports motivation, that is, boys may prefer the long jump that shows strength and speed, while girls prefer the rope skipping that shows rhythm and beauty. Even if there is the fact that “the preference for teaching themes affects the level of extracurricular sports motivation”, teacher of experimental group carefully organize the class and guide students to always maintain a positive interest in learning during intervention, for example, through group exercises by gender and creating auxiliary exercises similar to the long jump situation, pay attention to let students fully experience the complete technical actions, which has well activated the class atmosphere of the long jump; The creation of various forms of rope skipping exercises and sports games have well stimulated students’ interest in rope skipping learning and practice. In addition, teacher of experimental group organize a variety of physical activities and competitions in every class, the high learning momentum in PE then positively affects students’ extracurricular sports motivation.

#### Amount of extracurricular sports activities

Boys in the experimental group made significant progress at all stages, in contrast, the boys in the control group made no significant progress in test 2 to 3 and regressed in test 3 to 4. Girls in the experimental group made significant progress in test 2 to 3, test 3 to 4, and made progress in test 1 to 2, the girls in the control group made significant progress in test 2 to 3, and also made progress in test 3 to 4, the change from test 1 to 2 showed a downward trend. It can be seen from the above that the amount of extracurricular sports activities of students in the experimental group can maintain a stable growth during the intervention, but students in the control group show instability. In view of this, it is speculated that the teaching method may be the reason affecting students’ extracurricular sports participation. For example, boys in the control group regressed in test 3 to 4, the teaching theme in this period was rope skipping, teacher in the control group asked students to practice single rope skipping repeatedly around the rules of the middle school entrance examination, obviously, such teaching could not stimulate boys’ learning motivation, inhibit their extra- curricular sports activities motivation to a certain extent, and finally reduce their amount of extracurricular sports activities.

### The relationship between TCM variables of students in the experimental group

#### The influence of perceived need support on autonomous motivation in PE

The results show that the perceived need support of students in the experimental group can predict autonomous motivation in PE positively (*P* < .001). Teachers’ support behavior in class can positively affect students’ class motivation, which has been confirmed in many studies. Gracielle found that after receiving the training of autonomous support course, teachers have significantly improved the class motivation of students aged 12–14 after 8 months of autonomous support teaching [18]. Chang’s research found that teachers allowed students to decide the order of teaching projects, choose their own partners and provide students with group practice in unit teaching, after intervention of 6 weeks, the class motivation of sixth graders were significantly improved [19].

#### The influence of autonomous motivation in PE on autonomous motivation in LT

The results show that the autonomous motivation in PE of students can positively predict the autonomous motivation in LT. Hagger integrated multiple theory and put forward TCM, one of the key hypotheses is that students’ autonomous motivation in PE can be transferred outside the class [4], and this hypothesis has been confirmed by many relevant studies. For the junior school freshmen, their sports experience depend on the PE to certain extent, therefore, it is very important for teachers to create situations in PE to stimulate students’ motivation, because students’ autonomous motivation in PE may directly affect whether they are willing to participate in extracurricular sports activities.

#### The influence of autonomous motivation in LT on extracurricular sports participation intention

The research shows that among the three indirect paths of the mediation model, only the path RAI-LT → SN → INT has no significant indirect effect(*P*>.05), which also shows that motivation is more likely to affect intention through autonomous variables (attitude, perceived behavior control). In addition, autonomous motivation in LT can directly and effectively predict the intention of extracurricular sports participation(*P*<.01).TCM integrates the self-determination theory and the theory of planned behavior, they seem to be unrelated, but their combination has a certain theoretical basis. Chatzisarantis believes that individual beliefs will follow their motivation, so motivation will affect belief based social cognitive variables to a certain extent [20]. Hagger believes that belief based social cognitive variables have a specific function in TCM, that is, to explain the process from the satisfaction of basic psychological needs to the future demand satisfaction behavior. Based on the middle school students [21], Pihu found that in the parallel intermediary model, the indirect effect of RAI-LT → ATT → INT, RAI-LT → PBC → INT were significant, and the indirect effect of RAI-LT → SN → INT was not significant [22]. Tristan missed the data of subjective norm in the experiment, which made it impossible to weigh the effect of path RAI-LT → SN → INT, however, for the students aged 9–15, the effect of path RAI-LT → ATT → INT, RAI-LT → PBC → INT were significant, but the direct effect of the model (RAI-LT → INT) was not significant [23]. Different from the above studies, this study found that the direct effect of RAI-LT → INT is significant, that is, the effect of the parallel intermediary model belongs to partial intermediary.

#### The influence of extracurricular sports participation intention on extra- curricular sports behavior

The research shows that the intention of extracurricular sports participation of students can positively predict extracurricular sports behavior (*P*<.001), Hagger put forward TCM, one of the important assumptions is that the intention of extracurricular sports participation can positively predict extracurricular sports behavior [4]. Hagger’s survey of middle school students in Greece, Britain, Poland and Singapore shown that students’ intention of extracurricular sports participation can predict extracurricular sports behavior significantly and positively [24]. However, individual studies have found different results, for example, Viciana’s research on middle school students found that extracurricular sports participation intention can not effectively predict extracurricular sports behavior [25]. However, this study uses accelerometers to objectively measure the amount of extracurricular sports activities, which is different from previous studies (relevant studies use self-assessment scale). The unequal evaluation methods may also be one of the important reasons for the above results.

## Conclusion

The aim of this study is to examine the effect of the healthy physical education curriculum model on students’ extracurricular sports participation based on the TCM. In summary: The teaching of 12 weeks healthy physical education curriculum model has significantly improved the students’ perceived need support in PE, autonomous motivation in PE, autonomous motivation in LT and the amount of extracurricular sports activities. The structural characteristics of healthy physical education curriculum model provide need support for students’ learning, improve students’ autonomous motivation in and out of PE, and finally promote students’ participation in extracurricular sports.

## Data Availability

The datasets used and/or analysed during the current study are available from the corresponding author on reasonable request.
